# Genetic variants of the *oppA* gene are involved in metabolic regulation of surfactin in *Bacillus subtilis*

**DOI:** 10.1186/s12934-019-1176-z

**Published:** 2019-08-19

**Authors:** Xiaoyu Wang, Zhiyi Chen, Hui Feng, Xi Chen, Lihui Wei

**Affiliations:** Institute of Plant Protection, Jiangsu Academy of Agricultural Sciences, Nanjing, 210014 China

**Keywords:** Surfactin, *oppA*, Metabolomics, CCR, *ccpA*, Global regulator

## Abstract

**Background:**

*Bacillus subtilis* 916 has been identified as an effective biocontrol agent against *Rhizoctonia solani*, the causal pathogen of rice sheath blight, under greenhouse and field conditions. HPLC analysis showed that surfactin, a member of the lipopeptide family produced by *B. subtilis*, was the major antimicrobial substance.

**Results:**

Previously, we obtained a mutant strain of *B. subtilis* 916, Bs-H74, which produced significantly more surfactin than the wild type and presented 10% stronger inhibitory activity against *R. solani*. To explore the molecular mechanism underlying the higher surfactin productivity in the mutant, high-throughput proteomic analysis was carried out to analyze the differential protein expression. Our results showed that several differentially expressed proteins are involved in OppA, DegU and Carbon Catabolite Repression (CCR) regulatory pathways, which could be positively or negatively associated with surfactin biosynthesis. At both transcriptional and translational levels, we suggested that OppA may play a key role in surfactin synthesis regulation. Based on the above findings, we proposed the hypothesis that a point mutation in the *oppA* gene may lead to changes in oligopeptides acquisition in *B. subtilis*, and then the changed oligopeptides may activate or suppress the global regulatory protein, CcpA in the CCR pathway, and ComA and DegU may indirectly regulate surfactin synthesis in Bs-H74. To further explore the regulatory mechanisms in Bs-H74, metabolomics analysis was performed in this study. Interestingly, only 16 metabolites showed changes in abundance in Bs-H74 compared to Bs-916. Neohesperidin, a type of natural flavanone glycosides from citrus with a range of biological activities, increased by 18 times over the wild type Bs-916. This result implied exciting findings in regulatory mechanisms by OppA protein.

**Conclusions:**

In summary, this study has revealed the mechanisms underlying the improved antagonistic property with increased surfactin production in Bs-H74 at the gene, protein and metabolic levels, which may help to comprehend the map of the regulatory networks in *B. subtilis*. Findings from our work have provided a solid physical and theoretical basis for practically applying metabolic and genetic engineering to achieve improved and high-yielding biocontrol strains.

**Electronic supplementary material:**

The online version of this article (10.1186/s12934-019-1176-z) contains supplementary material, which is available to authorized users.

## Background

It is essential to control plant diseases for the abundance of foods, feeds and fibers for keeping up quickly growing global population. Agriculturists have been primarily dependent upon pesticides and fertilizers for maximal agricultural production. However, during recent years, people have changed their attitude towards pesticides and fertilizer due to the rising risks of ecological pollution, and the negative impact of pesticide residuals on human health and the earth ecosystem. Researchers have occupied their consideration towards the alternative of natural antagonistic microorganisms to control plant diseases. *Bacillus* species have abilities to duplicate quickly, become adaptive to different environmental conditions, and they also possess antagonism against a wide range of phytopathogenic microorganisms. *Bacillus* species produced volatile compounds to promote plant growth and activate plant defense by triggering the induced systemic resistance. Kloepper et al. [1] investigated the elicitation effect of different *Bacillus* species, such as *Bacillus subtilis*, *B. pumilus*, *B. cereus*, *B. pasteurii*, *B. amyloliquefaciens*, *B. mycoides*, and *B. sphaericus* on resistance induction in vegetable, *Arabidopsis* sp., loblolly pine and tobacco under field conditions and green house. Significant reduction in disease incidence and severity was observed in various diseases caused by bacteria, fungi, viruses, oomycetes and nematodes.

*Bacillus subtilis* strains produce a wind range of bioactive compounds that have awesome possibilities for horticultural and biocontrol applications. One in-depth study class is comprised of the peptide antibiotics and amphiphilic biosurfactants. Common examples from this class include surfactin [2–4], iturin compounds [5], and fengycin [6]. Surfactin is an extracellular peptide antibiotic, and its production in *B. subtilis* has been reported. Surfactin synthesis required the expression of *sfp* gene [7]. The *srfA* locus, an operon spanning over 25 kb, is required for the production of surfactin [8]. S*rfA* expression is induced at the end of exponential growth and is regulated by the products of two-component regulatory genes *comP* and *comA* and the *srfA* promoter region (P*srfA*) [9–13].

Because of the low yield of surfactin in *B. subtilis* strains, commercial production of surfactin has not been established. Attempts in optimizing the fermentation process have been made in recent studies to improve surfactin production [14–17]; however, these efforts failed to realize a commercially viable and profitable level of surfactin production. Some researchers have been focusing on screening for surfacing overproducing mutants or establishing genetically modified *B. subtilis* strains [18]. For example, recombinant strains with a modified promoter region of the *srfA* operon were constructed to induce *B. subtilis* to produce surfactin constitutively [19]; The *yngH* gene was overexpressed in *B. subtilis*, the surfactin production increased to 13.37 g/L [20]; A series of engineered strains with the modularization of metabolic pathways were constructed, the highest surfactin titer increased to 12.8 g/L [21]; Through the successful expression of *Vitreoscilla* hemoglobin gene, surfactin titer increased to 10.2 g/L [22]. Unfortunately, these measures had achieved limited success due to the complex intrinsic regulatory network underlying *srfA* expression [18, 19]. So far it remains difficult to produce surfactin in large quantities economically.

The oligopeptide transport system Opp is the significant and conserved transporter utilized by the *Bacillus* species and other microorganisms to import peptides. In the *B. subtilis*, *Opp* operon consists of five subunits—OppA, OppB, OppC, OppD, and OppF (Additional file 1: Figure S1). OppA protein plays an important role in the initial recognition and binding of substrates [23–26]. In addition to the combination of oligopeptides to provide amino acid nutrition to cells, some of OppA also play a role in other cellular activities, such as signal transduction, cell wall peptide metabolism, and as a molecular chaperone to assist in proteins fold in periplasmic space. Adrianne et al. reported that mutation of *opp* and *app* in *Clostridium difficile* seriously affected the expression of earlier sporulation genes and increased the amounts of spores. And *opp* and *app* mutants showed greater virulence in the hamster [27]. After OppA binds to the substrate, how to coordinate with other subunits to transport the substrate into the cell has not been reported in detail. So far, to the best of our knowledge, there is no report on the regulatory effect of Opp system on surfactin production.

Carbon Catabolite Repression (CCR) is a regulatory mechanism. CcpA is known as a central regulator of CCR in *B. subtilis*. After inactivation of *ccpA* gene, it can release the inhibition of α-amylase synthesis gene *amyE* by glucose [28–33]. CcpA binds to the catabolite-responsive element (*cre*) located in the transcription start region of *amyE*, does not require the cofactor Hpr. However, subsequent studies have shown that in most cases the combination of CcpA and *cre* sites requires cofactor protein P-(Ser)-HPr. The activity of P-(Ser)-HPr is affected by the ratio of intracellular ATP to inorganic phosphate (ATP/Pi), the intracellular concentration of 1,6-diphosphate fructose (FBP) and 6-phosphate glucose (G6P) [34, 35]. Many key enzyme genes in the glycolytic pathway are regulated by CcpA. Previous report have shown that activation of the *gap* operon is dependent on the presence of CcpA [30]. CcpA can directly or indirectly inhibit the expression of the citrate synthase gene *citZ* involved in the TCA cycle [36, 37]. In *Streptococcus gordonii*, CcpA influence the expression of adhesin genes, competence development and biofilm formation [27]. So far, the networks between CcpA and surfactin production has not been established.

*Bacillus subtilis* 916 was identified in a screening for biocontrol agent against the rice sheath blight caused by *Rhizoctonia solani*. It demonstrated its effectiveness in promoting plant growth and suppressing plant pathogenic organisms [38] in greenhouse and field tests. Previously, we obtained a mutant strain, Bs-H74, by means of low energy ion implantation in wild-type Bs-916 [39], which produces a significantly higher amount of surfactin and has better effects on controlling *R. solani*. High performance liquid chromatography (HPLC) analysis indicated that the major antagonistic substance produced by Bs-916 and Bs-H74 is surfactin, a member of the lipopeptide family [39]. In order to decipher the mechanisms underlying the higher productivity of surfactin in Bs-H74, in this study, we compared the sequences of P*srfA* and *comA* in Bs-H74 and the wild type Bs-916, and found that the sequences were identical. We further performed genomic, proteomic and metabolic analysis to explore new clues to the improved level of surfactin in Bs-H74. Results from this study may lead to a better understanding of the regulation of surfactin production, and therefore expedite the industrial production of surfactin and agricultural utilization of *Bacillus* spp. as biocontrol agents.

## Results and discussion

### ComA gene and *srfA* promoter sequence analysis

The *comA* gene and *SrfA* promoter (P*srfA*) were amplified from Bs-H74 and Bs-916 using primers listed in Table 4. P*srfA* and *comA* sequences were aligned by the clustW program. Our data showed that the P*srfA* and *comA* sequences were 100% conserved between the Bs-H74 and Bs-916 strains (data not shown). These results suggested that promotion in surfactin production in Bs-H74 may be due to other regulatory mechanisms.

### Identification of differentially expressed proteins

We compared the 2-DE profiles of soluble proteins from Bs-916 and Bs-H74 mutant strains and found 183 protein spots differed between the strains by twofold or more. 74 protein spots were identified by MS analysis and their complete peptide fingerprints were obtained. After excluding unknown or non-*Bacillus* proteins, 32 proteins were found to have decreased expression, and 15 proteins were found to have increased expression in the Bs-H74 mutant compared to the wild type Bs-916. BLAST searches against the NCBI nr database with Mascot revealed that these differentially expressed proteins may function in nucleosides, carbohydrates, and amino acids modification (Table 1). CitB, GapA RpoA, AcoL, PurA, FtsZ, PupG and GlnA protein were found to overabundant in the Bs-H74; FbaA, HutI and IolD protein were found to have decreased expression. As above 11 proteins were functionally annotated to CcpA regulation. PutC, IspA, RocA were found to overabundant in the Bs-H74; RocD proteins were found to have decreased expression. 4 proteins were functionally annotated to CodY regulation. OppA protein found to be overabundant were functionally annotated to ABC transporters.

**Table 1:** Identification of differentially regulated cellular proteins of *Bacillus subtillis*

Accession number	Gene	Description	P value	Fold change	Log.Fold change
16078991	DhaS	Aldehyde dehydrogenase	0.00	0.05	− 4.41
154687531	GapA	Glyceraldehyde-3-phosphate dehydrogenase, *ccpA* indirectly regulated	0.00	0.08	− 3.62
154687186	PckA	Phosphoenolpyruvate carboxykinase, *ccpN* repression	0.01	0.08	− 3.68
15616502	PutC	1-Pyrroline-5-carboxylate dehydrogenase, *codY* repression	0.00	0.10	− 3.31
16078863	CitB	Aconitate hydratase, *ccpA* regulated	0.00	0.12	− 3.11
52082083	DegU	Two-component regulator, *ccpA* regulated	0.01	0.09	− 3.50
34763277	RpoA	RNA polymerase alpha subunit, interact with the CcpA protein [47]	0.00	0.13	− 2.97
52080062	AcoL	Dihydrolipoamide dehydrogenase, *ccpA* regulated	0.02	0.10	− 3.39
16080734	AtpB	ATP synthase	0.02	0.14	− 2.83
154685773	MtnD	Acireductone dioxygenase, methionine salvage pathway	0.00	0.14	− 2.83
154684814	ProA	1-Pyrroline-5-carboxylate dehydrogenase, amino acid/nitrogen metabolism	0.00	0.14	− 2.83
154687206	PurC	Naphthoate synthase, nucleotide metabolism	0.05	0.11	− 3.21
154686652	PepT	Peptidase T (tripeptidase), utilization of peptides	0.00	0.19	− 2.43
37781613	SodA	SOD, important for survival of ethanol and paraquat stresses and at low temperatures	0.00	0.20	− 2.31
154687051	Pyk	Pyruvate kinase, glycolysis pathway	0.01	0.19	− 2.38
154687044	Mdh	Malate dehydrogenase, *ccpA* regulated	0.01	0.18	− 2.47
154686893	CymR	Pleiotropic regulator of sulfur metabolism, sulfur metabolism	0.02	0.18	− 2.48
154688140	PurA	Adenylosuccinate synthetase, interact with the CcpA protein [47]	0.01	0.24	− 2.06
154684785	YceD	Stress adaption protein	0.00	0.24	− 2.05
154685945	FtsZ	Cell division protein, interact with the CcpA protein [47]	0.09	0.16	− 2.63
63146095	IspA	Intracellular serine protease, *codY* repression	0.06	0.16	− 2.65
154686374	PupG	Purine nucleoside phosphorylase, *ccpA* regulated	0.01	0.24	− 2.07
194014533	PdhB	Pyruvate dehydrogenase, TCA cycle	0.00	0.26	− 1.96
154687585	ClpP	ATP-dependent Clp protease proteolytic subunit	0.00	0.24	− 2.04
154685670	Ndh	NADH dehydrogenase (menaquinone 7 and no proton), respiration	0.00	0.27	− 1.89
154687897	RocA	1-Pyrroline-5-carboxylate dehydrogenase, *codY* repression	0.05	0.22	− 2.17
16080001	Tpx	Putative peroxiredoxin, spx regulated	0.00	0.25	− 2.03
154688008	PepT	Peptidase T, amino acid/nitrogen metabolism	0.00	0.30	− 1.74
154687826	YwjH	Putative translaldolase, pentose phosphate pathway	0.01	0.32	− 1.63
216396	GlnA	Glutamine synthetase, interact with the CcpA protein [47]	0.05	0.20	− 2.35
154687185	MetK	*S*-Adenosylmethionine synthetase, methionine salvage pathway	0.08	0.21	− 2.22
16078208	OppA	Oligopeptide ABC transporter, uptake of peptides/*codY* repression	0.01	0.24	− 2.05
221311046	GbsR	Regulation of osmoprotection	0.14	5.27	2.40
154685807	DnaK	Chaperone protein	0.00	3.27	1.71
154687392	FadA	Acyl-CoA acetyltransferase, fatty acid degradation	0.00	4.71	2.24
52081769	FadE	Acyl-CoA dehydrogenase, fatty acid degradation	0.01	5.44	2.44
154686777	IspH	Non-mevalonate pathway, biosynthesis of lipids	0.04	5.32	2.41
154688074	IolD	Formation of 5-deoxy-d-glucuronic acid, *ccpA* regulated	0.04	5.50	2.46
154684631	TufA	Elongation factor Tu	0.01	5.42	2.44
154688042	HutI	Imidazolonepropionase, *ccpA* regulated	0.00	4.47	2.16
154688124	RocD	Omithine-oxo-acid transaminase, *codY* repression	0.01	4.74	2.24
154685878	PdhD	Dihydrolipoamide dehydrogenase, TCA cycle	0.01	6.07	2.60
154687798	AtpA	ATP synthase subunit	0.04	10.98	3.46
154687827	FbaA	Fructose-bisphosphate aldolase, interact with the CcpA protein [47]	0.00	9.73	3.28
1389732	MetE	Methionine synthetase, methionine salvage pathway	0.12	20.94	4.39
154686979	SdhB	Succinate dehydrogenase, TCA cycle	0.07	26.59	4.73
154685475	YhfE	Putative endoglucanase	0.02	35.31	5.14

### Functional annotation of proteins

The overrepresentation of GO categories among the 47 proteins showing a relative change in abundance in Bs-H74 mutant was assessed using BiNGO. The Bs-H74 mutant has modulated the abundance of proteins functionally related to transcription, translation and various metabolic processes, such as small molecules formation and RNA processing (Table 1). Protein functional annotation against KEGG Orthology (KO) showed that several proteins were overabundant in the Bs-H74 mutant in comparison to the wild type. According to the proteome data and reference, these proteins were categorized into 3 subgroups: (i) carbon catabolite repressors and their regulators, (ii) ABC transporters, (iii) transcription factors and their regulators.

**Fig. 1:**
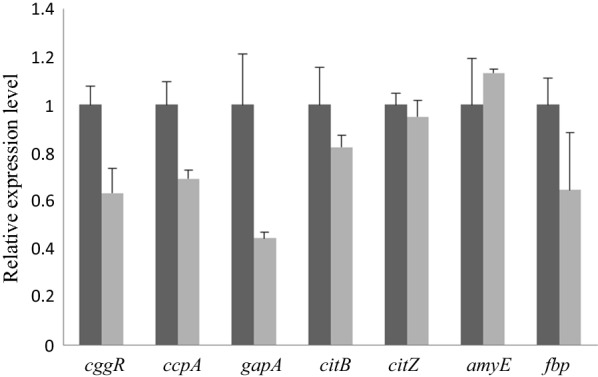
Expression of CCR-associated genes in Bs-H74 and Bs-916. Grey column indicated wild type Bs-916 strain; black column indicated Bs-H74 strain

Expression levels of several proteins involved in Carbon Catabolite Repression (CCR) aroused our attention. GapA, glyceraldehyde 3-phosphate dehydrogenase, expressed more than threefold in Bs-H74. RT-PCR was used to determine the transcription levels of *cggR*, *ccpA* and *gapA* in both the wild type Bs-916 and the mutant Bs-H74 strains. The five genes, *cggR*, *ccpA*, *fbp*, *citB* and *gapA* were upregulated in the H74 strain (Fig. 1). *AmyE* gene was downregulated, *citZ* gene was minor upregulated. *Fbp*, *cggR* and *amyE* gene expression were consistent with previous reported result. But *gapA*, *citZ* and *citB* gene expression were not consistent with previous reported result [24, 30, 32, 33]. Our explanation was that *oppA* gene not only regulate the expression of CCR-associated genes, but also regulate the expression of GRP, global regulator protein, for example, *comA* gene in Bs-H74. We suggested that regulatory mechanisms of OppA protein is complicated, further study may be carried out to elucidate the mechanisms.

**Fig. 2:**
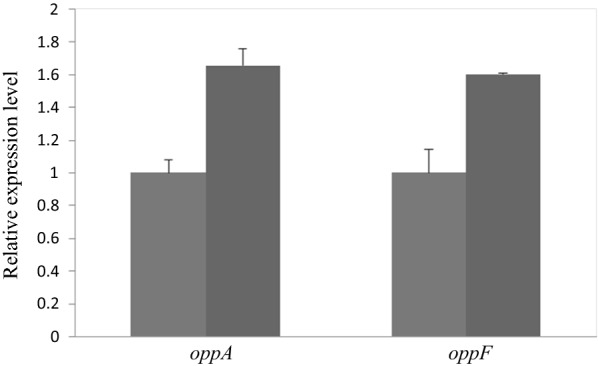
Expression of *opp* system genes in Bs-H74 and Bs-916. Grey column indicated wild type Bs-916 strain; black column indicated Bs-H74 strain

Another upregulated protein is the OppA. The expression level of OppA increased by more than twofold in Bs-H74. Real-time-PCR further identify that transcription levels of *oppA* is upregulated. And *oppF*, belonging to the Opp operon, as we predicted, is also upregulated (Fig. 2). Transcriptional regulatory protein DegU is a member of the two-component regulatory system DegS/DegU, which plays an important role in the transition growth phase. Expression level of DegU increased by more than sevenfold in Bs-H74.

### Resequencing and mutations detection

In this study, we found 8 non-synonymous mutations resulting from SNPs, deletions, insertions, and shifts in Bs-H74. Six of the non-synonymous mutations led to mutated genes that coded for OppA/AppA oligopeptide transport protein family, transcriptional repressor of sporulation, collagen-like protein, proline permease, and glycerol-l-phosphate dehydrogenase. Specifically, in this study, all SNPs resulted in amino acid substitutions. As we expected, one substitution, Trp for Arg, occurred in the OppA protein (Table 2). This mutation may be responsible for the up-regulated expression of OppA in Bs-H74.

**Table 2:** Validated mutations in Bs-H74

Gene	Mutation	Gene function
*RBAM_007770*	V209A (GTA → GCA); V230L (GTA → TTA); I239V (ATA → GTA)	Collagen-like protein, colony and biofilm formation
*oppA*	W519R (TGG → CGG)	Oligopeptide transport protein
*rapA*	P259L (CCG → CTG)	Transcriptional repressor of sporulation, control of sporulation initiation
*araM*	Q226K (CAG → AAG)	Glycerol-1-phosphate dehydrogenase, biosynthesis of phosphoglycerolipids
*RBAM_005790*	C → T intergenic (+ 390/− 228)	Putative transcriptional repressor of sporulation and degradative enzymes production
*czcD*	+ 38 bp intergenic (− 6/− 187)	Cation efflux, resistance against Zn, Cu, Co, Ni
*ycgO*	I238N (ATC → AAC)	High affinity proline permease, proline uptake
*nasD*	E307D (GAA → GAC)	Utilization of nitrite as nitrogen source

### Metabolite identification

In this study, metabolomics analysis resulted in the detection of 16 metabolites that showed changes in abundance in Bs-H74 compared to the wild type (Table 3). Neohesperidin, benzoylformic acid, oxamic acid, glucoheptonic acid, *N*6-acetyl-l-lysine, 4-hydroxyphenylacetic acid, 5′-methylthioadenosine, phenylpyruvate, 2-hydroxy-3-isopropylbutanedioic acid, and glycolic acid were found to be increased in abundance in Bs-H74. 2,4,6-trihydroxybenzophenone, conduritol b epoxide, 5-methoxytryptamine, lactulose, and monopalmitin were found to be decreased in abundance in Bs-H74. These metabolites are involved in amino acids metabolism, biosynthesis of secondary metabolites, biosynthesis of antibiotics, biosynthesis of amino acids, lipids and lipid-like molecules metabolism, and carbohydrates metabolic pathways, respectively.

**Table 3:** Metabolites showing changes in abundance in Bs-H74 compared to Bs-916

Metabolite	KEGG name	Formula	Fold	KEGG
2,4,6-Trihydroxybenzophenone	2,4,6-Trihydroxybenzophenone	C13H10O4	− 1.06	C06356
Conduritol b epoxide 2	Conduritol b epoxide 2	ND	− 0.71	ND
5-Methoxytryptamine 1	5-Methoxytryptamine	C11H14N2O	− 0.55	C05659
Lactulose 1	Lactulose	C12H22O11	− 0.52	C07064
1-Monopalmitin	1-Monopalmitin	ND	− 0.22	ND
Glycolic acid	Glycolic acid	C2H4O3	0.4	C00160
11-Beta-prostaglandin-F-2-alpha 1	11-epi-Prostaglandin F2alpha	C20H34O5	0.42	C05959
2-Hydroxy-3-isopropylbutanedioic acid	2-Hydroxy-3-isopropylbutanedioic acid	ND	0.45	ND
Phenylpyruvate	Phenylpyruvate	C9H8O3	0.5765	C00166
5′-Methylthioadenosine 1	5′-Methylthioadenosine	C11H15N5O3S	0.79	C00170
4-Hydroxyphenylacetic acid	4-Hydroxyphenylacetic acid	C8H8O3	1.009	C00642
*N*-Epsilon-Acetyl-l-lysine 1	*N*6-Acetyl-l-lysine	C8H16N2O3	1.728	C02727
Glucoheptonic acid 1	Glucoheptonic acid		2.03	
Oxamic acid	Oxamic acid	C2H3NO3	2.58	C01444
Benzoylformic acid 3	Benzoylformic acid	C8H6O3	3.09	C02137
Neohesperidin	Neohesperidin	C28H34O15	18.826	C09806

Neohesperidin was found to have increased by 18 times in abundance in Bs-H74 compared to Bs-916 (Table 3). Previous research showed that Neohesperidin is a type of natural flavanone glycosides from citrus with a range of biological activities. It was first discovered in plants and usually distributed in wild orange, citrus aurantium, immature grapefruit. Neohesperidin activated AMP-activated protein kinase (AMPK) by phosphorylation and increased glucose consumption in HepG2 cells. These results indicated that Neohesperidin has a potential diabetes prevention and treatment effect [40]. Neohesperidin has also antibacterial, anti-allergic and anti-tumor pharmacological activities [41–43].

Glucoheptonic acid was found to have increased by two times in Bs-H74 than Bs-916. Glucuronic acid is a uronic acid in which C-6 hydroxyl group of glucose is oxidized to a carboxyl group. D-glucuronic acid generally is present as stable 3,6-lactone of the furan ring. d-glucopyranuronic acid is present in the oligosaccharide at the junction of the glycosaminoglycan chain, heparin and chondroitin.

The content of 5′-methylthioadenosine (MTA) was found to be increased in Bs-H74. MTA is a metabolite in the metabolism of polyamines and methionine. The molecular structure was reported in 1924. Precursor is *S*-adenosylmethionine. MTA promotes apoptosis of liver cancer cells and inhibits apoptosis of normal liver cells.

The content of *N*6-acetyl-l-lysine and glycolic acid increased in Bs-H74. Glycolic acid is the first member of the a-hydroxy acid family of carboxylic acids. It is used in various skin-care products. Biological production of glycolic acid requires glyoxylic acid circle. As intermediates, oxaloacetic acid and glyoxylic acid are aminated to generate aspartic acid and glycine, respectively.

### Diagram of a proposed OppA regulatory pathway for surfactin biosynthesis

**Fig. 3:**
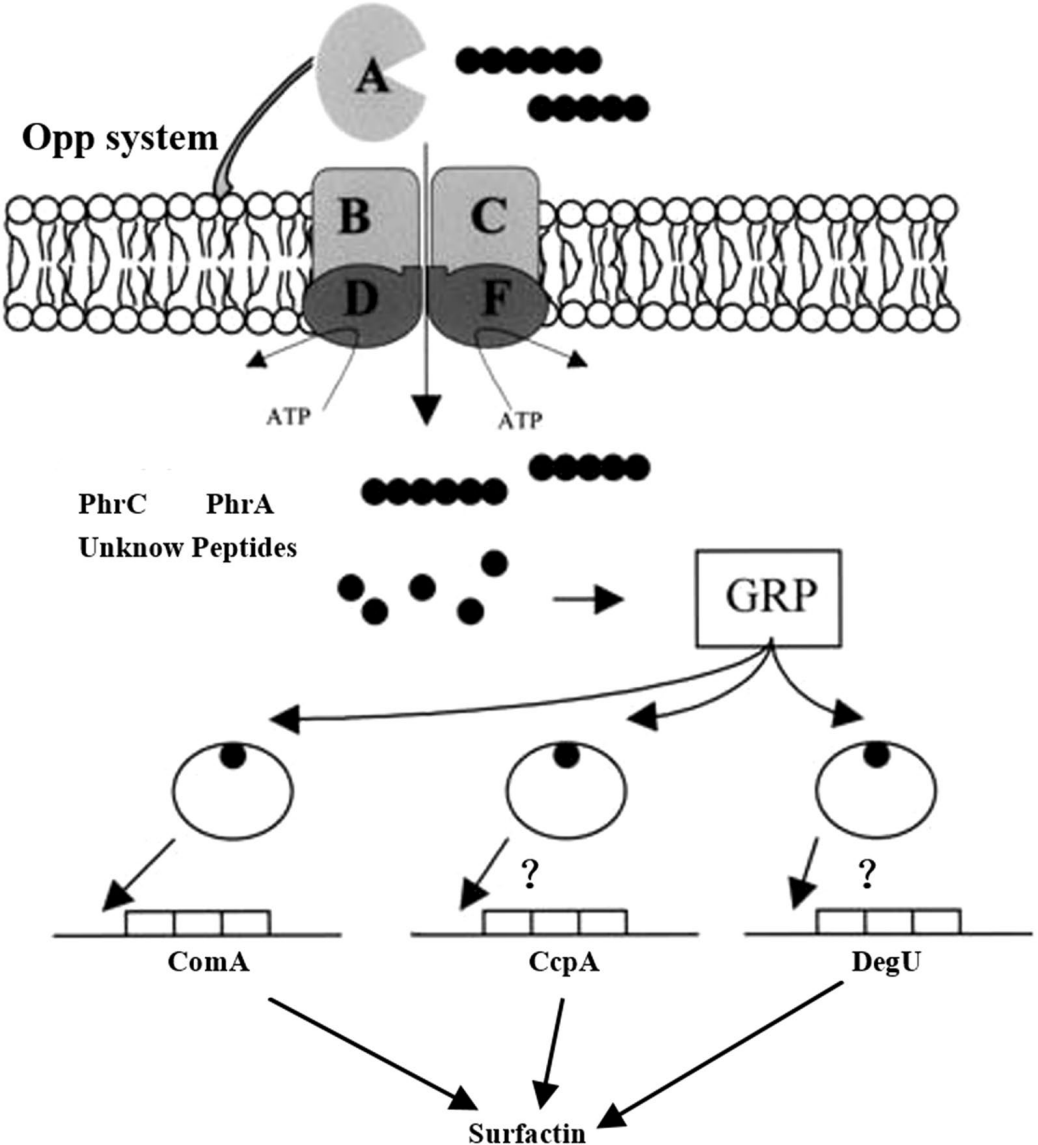
Proposed diagram of Opp regulation of surfactin synthesis. Opp mediates the uptake of peptides, such as PhrA, PhrC and unknown peptides as a nutrient source. The acquisition of peptides indirectly influences *comA*, *ccpA* and *degU* gene expression through an unknown mechanism, which may activate surfactin synthesis gene expression. GRP, global regulator protein, Black arrows, putative regulatory effects, Black arrows with question mark, unclear or indirect effects

In terms of signal transduction, OppA has two possible modes of action: one is the binding of non-specific oligopeptide signal molecules, transporting them into the cell, activating the corresponding transcriptional regulatory factors by changing the intracellular amino acid concentration, and directing the downstream cellular metabolic activity; the second is the combination of specific oligopeptide signal molecules secreted by the bacteria to the outside, transmitting the signal into the cell, acting on the expression of the target gene, regulating cellular metabolic activities such as spore formation, splicing and pathogenicity [44–46]. According to the findings from this study, we proposed the hypothesis that mutations in the *OppA* gene may lead to the changes in oligopeptides acquisition. In other words, the mutated OppA protein may combine different types or different amounts of oligopeptides. Then the changed oligopeptides may activate or suppress the global regulator protein CcpA in CCR pathway. ComA and DegU may indirectly regulate surfactin synthesis in Bs-H74 mutant strain (Fig. 3).

*Bacillus subtilis* has been intensively studied for biological control of plant diseases. Previously, a mutant strain Bs-H74 demonstrated a 10% stronger inhibitory activity against *R. solani*, the causal pathogen of rice sheath blight than the wild type Bs-916. HPLC analysis showed that Bs-H74 produces significantly more surfactin, a major antimicrobial substance. The *srfA* locus and ComA are required for the production of surfactin, but our data showed that the P*srfA* and *comA* sequences were 100% conserved between the Bs-H74 and Bs-916 strains. These results suggested that promotion in surfactin production in the mutant may be due to other regulatory mechanisms.

To explore the underlying molecular mechanisms of the higher surfactin production in the mutant, high-throughput proteomic analysis was carried out to compare the protein expression between Bs-916 and Bs-H74. Proteome data showed that OppA, DegU and proteins in Carbon Catabolite Repression (CCR) may be involved in surfactin production. In order to test this hypothesis, we verified the transcription of the *opp* system genes. Data showed that both the *oppA* and *oppF* genes were upregulated in the Bs-H74 strain. The expression levels of CCR-associated genes are different between the wild type Bs-916 and the mutant Bs-H74. The central regulated gene of CCR, *ccpA* gene was upregulated in the Bs-H74 strain as we predicted. These results showed that *oppA* and *ccpA* may be involved in surfactin production regulatory pathways. Through the SPINE (Strep–protein interaction experiment) method, 44 functionally known proteins that may interact with the CcpA protein were identified. Among these proteins, 7 proteins appear in our proteome data, which are RpoA, FtsZ, PurA, FbaA, Mdh, CitB, GlnA. In addition, SrfAA, SrfAB, SrfAC proteins are also included in these proteins. However, the authors did not further analyze the interaction between CcpA protein and SrfAA, SrfAB, and SrfAC proteins. They provided the hypothesis that CcpA proteins interactive with RpoA and CodY by activating *ackA* (the gene encoding acetate kinase). Finally, RpoA and CodY proteins and CcpA can forms complexes, which regulate the upstream sequence of the *ackA* promoter and activate gene transcription [47]. In this study, RpoA protein expression was up-regulated, consistent with the positive regulation of CcpA in the above studies. We also detected up-regulated expression of SrfAA, SrfAB, and SrfAC by RT-PCR, which implicated that CcpA may be positively regulate the expression of the *srf* operon. However, it is unclear that CcpA protein activates the synthesis of surfactin by directly binding to the *srf* operon, or indirectly by binding to the relevant factors of surfactin synthesis. Further studies on protein interaction will be carry out to clarify.

Transcriptional regulatory protein DegU is partner of the two-component regulatory system DegS/DegU which plays a critical role in the exponential growth period. DegS/DegU involved in the control of many genes expression of different biological processes, for example, biofilm formation, flagellum formation and competence for DNA uptake, positively or negatively regulates expression of many different genes. The phosphorylated form of DegU is important to synthesis of degradative enzymes, flagellum formation and biofilm formation. *SrfA* expression has been known to be activated by DegU-P, as the mutation of *degU* causes downward expression of *srfA* gene in *B. subtilis*. DegU is also regulated indirectly by RghR. RghR represses Rap proteins by protein–protein interactions, which inhibit the binding of DegU to DNA, causing to inhibition of *srfA* expression [48, 49]. In the present study, we found that the increased transcription/expression of DegU was associated with the higher production of surfactin, which is consistent with previous studies.

And then we found a point mutation in the *oppA* gene in Bs-H74 through whole-genome resequencing. This SNP mutation is not located in “Venus’s flytrap”, suggesting that other regulatory mechanisms may exist. In previous studies on *C. difficile*, the *opp* operon was reported to be repressed by the important regulators CcpA, CodY and glucose, implying that the Opp transporter is involved in adjustment to supplement restricting conditions [50]. In *B. subtilis*, CcpA can bind directly to upstream region of the *degU*, implying that CcpA may be interacted with *degU* by protein and DNA [51]. In this study, we detected that *ccpA* and *degU* genes were upregulated in the Bs-H74 strain implying that the two global regulatory proteins may be involved in *oppA* signaling pathway.

The biosynthesis of surfactin is catalysed through NRPS, initiated from the condensation of fatty acids and Glu. Other constituent amino acids, Asp, Leu, Val are assembled through the NRPS multi-enzyme complex, comprising adenylation, condensation, and thiolation domains responsible for the activation of amino acids and peptide chain elongation. The content of *N*6-acetyl-l-lysine and glycolic acid were found to be increased in Bs-H74. As intermediates, oxaloacetic acid and glyoxylic acid are aminated to generate Asp and Gly, respectively. In our study, MTA increased by 0.8-fold. It is involved in the methionine salvage pathway. This pathway regulate some important metabolites, for example, *S*-adenosylmethionine (SAM). MTA is produced during polyamine synthesis, which is a suppressor of polyamine synthesis and transmethylation reaction. In the proteomic data of this study, the related proteins are detected, MetK, MetE and MtnD. Among them, MetK catalyze the reaction of methionine to form SAM, is up-regulated. So the increase of SAM content leads to an increase in MTA content. Shigeo Tojo et al. reported that the *mthA* gene point mutant was responsible for overproduction of bacilysin. This mutant increase in the SAM level was directly responsible for bacilysin overproduction, as confirmed by overexpression of the *metK* gene encoding SAM synthetase [52]. Based on these data, additional amino acids and SAM may be used to produce surfactin and provide a reasonable explanation. In this study, in addition to surfactin production increase, another unknown secondary metabolite, Neohesperidin-like flavonoid compound was synthesized in large quantities. In *Bacillus*, the KEGG pathway indicates that the synthesis of flavonoids involves phenylalanine metabolic pathway, which catabolite of pathway provide precursors for flavonoids synthesis. Metabolomics data showed that the related metabolite phenylpyruvate was increased increased by more than twofold. And SAM was frequently a methyl donor in the synthesis of flavonoids.

## Conclusion

Subsequently, we proposed the hypothesis that the point mutation in the *oppA* gene may lead to changes in oligopeptides acquisition in *B. subtilis*, and then the changed oligopeptides may activate or suppress the global regulatory protein, CcpA in CCR pathway, and ComA and DegU may indirectly regulate surfactin synthesis in Bs-H74 mutant strain.

In our study, one residue has been mutated to a substitution, Trp for Arg in OppA protein. This SNP, W519R (TGG → CGG), is not a previously reported active site, which may be the key amino acid site that can change the regulating process. At both the transcriptional and translational levels, we suggested that OppA plays a key role in surfactin synthesis regulation. To further explore the regulatory mechanism in Bs-H74, metabolomics studies were performed in this study. Interestingly, only 16 metabolites showed changes in abundance in Bs-H74 compared to Bs-916. It was found that metabolites related to amino acids metabolism, biosynthesis of secondary metabolites, biosynthesis of antibiotics, biosynthesis of amino acids, lipids and lipid-like molecules metabolism, and carbohydrates metabolic pathways were enhanced in the Bs-H74 mutant strain. Neohesperidin, a natural flavanone glycoside from citrus with a range of biological activities, increased by 18 times in Bs-H74 than the wild type Bs-916. The mutation in the *oppA* gene may have caused significant changes of many genes at transcriptional and translational levels, while only one or two metabolites have greatly increased their abundance. It suggested that the upstream regulation has enriched some certain metabolic pathways, thus increased the production of surfactin and a few other metabolites, such as Neohesperidin.

In summary, with the combination of the multi-omics data from this study and the information from previous research, we have identified the potential genes and regulators that could be positively or negatively regulating *srfA* expression. These findings have revealed the mechanism underlying the improved antagonistic property with increased surfactin production in Bs-H74. This study has provided a solid physical and theoretical basis for practically applying metabolic and genetic engineering to achieve improved and high-yielding biocontrol strains.

## Materials and methods

### Strain and culture media

*Bacillus subtilis* strains Bs-916 and mutant Bs-H74 was obtained and maintained in our lab [39]. Luria–Bertani (LB) medium was made as previously described [53]. *B. subtilis* strains were grown at 28 °C in either YPGA medium or YPG liquid medium with an agitation rate of 130 rpm. A single colony from YPGA solid media was transferred into YPG liquid media and then incubated for 24 h at 28 °C in a shaker.

### Protein extraction

Protein extractions were performed according to Faurobert et al. with minor modification [54]. Samples were ground to fine powder with liquid nitrogen, then suspended in 3 volumes of extraction buffer (700 mM sucrose, 500 mM Tris, pH 8.0, 100 mM KCl, 2% (v/v) β-mercaptoethanol, 2 mM phenylmethylsulfonyl fluoride, pH 8.0) and incubated for 20 min on ice. Afterward, an equal volume of Tris-saturated phenol was added. Samples were shaken on ice for 20 min and then centrifuged (15 min, 5000*g*, 4 °C). The phenolic phase was recovered and re-extracted with the same volume of extraction buffer. Subsequently, centrifugation was repeated and 4 volumes of precipitation solution (0.1 M ammonium acetate in methanol) were added to the recovered phenol phase. Protein was precipitated at − 20 °C overnight. After centrifugation (15 min, 5000*g*, 4 °C), the protein pellet was washed twice with the precipitation solution and once with cold acetone. Protein pellet was lyophilized by vacuum, and stored at − 80 °C until use. Before 2-DE, protein pellets were dissolved in a lysis buffer (8 M urea, 2 M thiourea, 4% CHAPS, 13 mM DTT, 2% pharmalyte 3–10) and used immediately after debris was removed by centrifugation as described above. The protein concentration was determined with Bio-Rad protein assay kit based on the Bradford method using BSA as standard. Three independent protein extractions were performed.

### 2-DE and image analysis

Samples were applied to 17 cm liner IPG strips (pH 4–7). After 14-h rehydration, isoelectric focusing was performed on PROTEAN IEF Cell (Bio-Rad) as following program: 30 min at 250 V, 1 h at 1000 V, 5 h to increase the voltage from 1000 to 8000 V, 7.5 h at 8000 V. Gels were then subjected to 2 × 20 min equilibration, using buffer I and buffer II, which basically contains 6 M Urea, 50 mM Tris–HCl (pH 8.8), 30% glycerol, 2% (w/v) SDS, with additional 2% (w/v) DTT (I) and 2.5% (w/v) iodoacetamide(II) respectively. SDS-PAGE was performed with 12% acrylamide gels in the PROTEAN MINI 2 (Bio-Rad) for 5 h at 180 V. PI and molecular mass of the protein were determined respectively by the liner pH arrangement of IPG strips and SDS-PAGE marker. Proteins were visualized with Coomassie Brilliant Blue (CBB) G250. Protein spots were detected by PDQuest software (Bio-Rad) and normalized to total quantity in valid spots under PPM (× 1,000,000) scaling step. According to the PDQuest software, spot quantity is the total intensity of a defined spot in a gel image. The intensity is the sum of intensities of the image pixels inside a boundary. It is calculated during spot detection and Gaussian fitting, which is calculated by PDQuest software. After the manual process, the well-separated spots in all triplicate gels were analyzed by Students’ T test (P < 0.05).

#### Tryptic digestion and protein identification

Protein spots were excised from gels, destained for 20 min in 30 mM potassium ferricyanide/100 mM sodium thiosulfate (1:1 v/v) and washed in Milli-Q water until the gels were destained. The spots were kept in 0.2 M NH_4_HCO_3_ for 20 min and then lyophilizated. Each spot was digested overnight in 2 µL 12.5 ng/µL trypsin in 0.1 M NH_4_HCO_3_. The peptides were extracted with 50% ACN, 0.1% TFA three times. All mass spectra were acquired on an AutoFlex MALDI-TOF/TOF mass spectrometer with LIFT technology (Bruker Daltonics, Bremen, Germany). Tryptic digests were prepared on AnchorChip sample plate (Bruker Daltonics, Bremen, Germany) according to the manufacturer’s instructions. MS/MS data were acquired with a N_2_ laser at 25-Hz sampling rate. The data set was submitted to MASCOT for protein identification. National Center for Biotechnology non-redundant (NCBInr 20080221) protein database (6122577 sequences; 2096230148 residues) was searched against. The search was performed using green plants as taxonomy, which contained 473596 sequences. Other parameters for searching were enzyme of trypsin, one missed cleavage, fixed modifications of carbamidomethyl (C), and variable modifications of oxidation (Met). Peptide tolerance of 100 ppm, fragment mass tolerance of ± 0.5 Da, and peptide charge of 1 + were selected. Only significant hits, as defined by the MASCOT probability analysis (P < 0.05), were accepted.

### Functional annotation of proteins

Overrepresentation of gene ontology (GO) terms for differentially abundant proteins was calculated with the Biological Network Gene Ontology Tool (BiNGO v. 2.44 [55]) and visualized in Cytoscape [56]. The hypergeometric test used for the enrichment analysis in BiNGO was performed using a FDR-corrected P-value cutoff of 0.05 and using all GO annotated genes of Bs168 as a background reference. The annotation was done using the Basic Local Alignment Search Tool for proteins (BLASTp) with default settings.

### Real-time PCR

Quantitative real-time RT-PCR analysis was performed by ABI Stepone plus using SybrGreen (Sangon Ltd) technology on ABI Stepone plus (Applied Biosystems, Foster City, USA). RNA was extracted by RNAprep pure kit (TIANGEN Ltd, Beijing, China). One to two microliters of RNA solution was digested with DNase I (TaKaRa Biotech. Co. Ltd, Dalian, China) and used in the RT-PCR analyses. RT was initiated in the presence of oligo-(dT) primers (42 °C, 30 min), and, after inactivation of the reverse transcriptase (95 °C, 5 min), the appropriate primers (Table 4) were added for PCR cycling (3 min at 95 °C, 40 cycles of 7 s at 95 °C, 10 s at 57 °C, and 15 s at 72 °C). Amplification of a constitutively expressed gene (*16sRNA*) served as an internal control in the RT-PCR assays.

**Table 4:** Oligonucleotide sequences of *comA*, *PsrfA* and CCR-associated genes

Oligonucleotide	Oligonucleotide sequences
*comA*	5′ ATGAAAAAGATACTAGTGATTGATG 3′ 5′ TTAAAGTACACCGTCTGATTTC 3′
P*srfA*	5′ AAAATGTCATGAAAGAATCGTTGTAAG 3′ 5′ CGCAAGATTTGAAATGCTCGTGTGA 3′
*ccpA*	5′ CAAAAGTCTCATCGCCACG 3′ 5′ CGAGATTGAGCCTGATGGTC 3′
*cggR*	5′ ATCAGGCTTACCGAACCGA 3′ 5′ GCGTCAACCCTAAAACATCC 3′
*citB*	5′ ACCGTTGTTGCGGCAGTAT 3′ 5′ TCCTGGTGTTGCTGAGCTTC 3′
*gapA*	5′ CATTCCTACATCAACTGGTGCTG 3′ 5′ CATTCCTACATCAACTGGTGCTG 3′
*fbp*	5′ ATAACGGCAACCTGCTGATT 3′ 5′ GTATTCGCCTGTCCATAAGTACC 3′
*16sRNA*	5′ GAGGCAGCAGTAGGGAATCTT 3′ 5′ CCGTGGCTTTCTGGTTAGGT 3′

### Resequencing

Genomic DNA (5 μg) isolated from single colonies of the endpoint strains was used to generate the genomic DNA library using the Illumina genomic DNA library generation kit following the manufacturer’s protocol (Illumina Inc., San Diego, CA, USA). Briefly, bacterial genomic DNA was fragmented by nebulization. The ends of fragmented DNA were repaired by T4 DNA polymerase, Klenow DNA polymerase, and T4 polynucleotide kinase. The Klenow exo minus enzyme was then used to add an ‘A’ base to the 3′ end of the DNA fragments. After the ligation of the adapters to the ends of the DNA fragments, the ligated DNA fragments were subjected to 2% 1 × TAE agarose gel electrophoresis. DNA fragments ranging from 150 to 300 bp were recovered from the gel and purified using the Qiagen mini gel purification kit. Finally, the adapter-modified DNA fragments were enriched by PCR. The final concentration of the genomic DNA library was determined by Nano drop and validated by running 2% 1 × TAE agarose gel electrophoresis. A 4 pM genomic DNA library was used to generate the cluster on the Flowcell following the manufacturer’s protocol. The genomic sequencing primer v2 was used for all DNA sequencing. A 36 cycle sequencing run was carried out using the Illumina analyzer following the manufacturer’s protocol.

### The whole-genome sequencing and mutation detection

Bs-H74 mutant strain was sequenced on Illumina GA, GA II, and GA IIx instruments. The resulting reads were obtained. Reads were mapped to the reference genome of the Bs-916 strain (GenBank accession no. AFSU00000000 [57]), and mutations were predicted using the breseq computational pipeline [58]. This pipeline detects point mutations, deletions, and new sequence junctions that may indicate IS-element insertions or other rearrangements, as described in its online documentation. Large duplications and amplifications were predicted manually by examining the depth of read coverage across each genome.

### Sample preparation for metabolite identification

Samples were taken as quickly as possible from the fermentor and immediately quenched to halt cellular metabolism, at − 45 °C in 0.4 mL methanol extraction liquid *(V methanol: V chloroform *= 3:1). 20 μL of l-2-Chlorophenylalanine was added as an internal standard to the sample. The intracellular metabolites were extracted from the cell suspension with a ball mill. Samples were homogenized in a ball mill for 4 min at 45 Hz, ultrasound treated for 5 min (incubated in ice water) for 5 times, and then centrifuged for 15 min at 12,000 rpm at 4 °C. The supernatant was transferred (0.39 mL) into a fresh 2 ml GC/MS glass vial. 15 μL of supernatant from each sample was pooled as QC sample. Cell extracts were dried in a vacuum concentrator without heating. The dried extracts were derivatized with 20 μL of methoxy amination hydrochloride (20 mg/mL in pyridine) for 30 min at 80 °C. Subsequently, the extracts were silylated for 2 h at 70 °C with 30 μL of the BSTFA regent (1% TMCS, v/v). 10 μL of FAMEs (Standard mixture of fatty acid methyl esters, C8–C16:1 mg/mL; C18–C24:0.5 mg/mL in chloroform) was added to the QC sample. After samples cooled down to the room temperature, they were mixed thoroughly for GC-TOFMS analysis.

### GC–TOFMS analyses for metabolite identification

GC–TOFMS analysis was performed using an Agilent 7890 gas chromatograph system coupled with a Pegasus HT time-of-flight mass spectrometer. The system utilized a DB-5MS capillary column coated with 5% diphenyl cross-linked with 95% dimethylpolysiloxane (30 m × 250 μm inner diameter, 0.25 μm film thickness; J&W Scientific, Folsom, CA, USA). A 1 μL aliquot of the analyte was injected in splitless mode. Helium was used as the carrier gas, the front inlet purge flow was 3 mL/min, and the gas flow rate through the column was 1 mL/min. The initial temperature was kept at 50 °C for 1 min, then raised to 310 °C at a rate of 20 °C/min, then kept for 5 min at 310 °C. The injection, transfer line, and ion source temperatures were 280, 270, and 220 °C, respectively. The energy was − 70 eV in electron impact mode. The mass spectrometry data were acquired in full-scan mode with the m/z range of 50–500 at a rate of 20 spectra per second after a solvent delay of 455 s.

Chroma TOF 4.3X software of LECO Corporation and LECO-Fiehn Rtx5 database were used for raw peaks exacting, the data baselines filtering and calibration of the baseline, peak alignment, deconvolution analysis, peak identification and integration of the peak area. The RI (retention time index) method was used in the peak identification, and the RI tolerance was 5000. Remove metabolic features detected in < 50% of QC samples.

## Additional file

**Additional file 1: Figure S1.** Opp system in *Bacillus*, oligopeptide ABC transporter family. A, an extracellular ligand-binding lipoprotein (OppA), two transmembrane proteins (OppBC) that form a membrane-spanning pore, and two cytoplasmic ATPases (OppDF) that drive the transport of the peptide into the cell; B, 3D structure of OppA protein; C, Identification of oligopeptide transporter systems in Bs-916.
