# Supplementary material for: Genetic variants of the oppA gene are involved in metabolic regulation of surfactin in Bacillus subtilis
Source: Microb Cell Fact. 2019 Aug 19;18:141. doi: 10.1186/s12934-019-1176-z (PMC6699124; doi:10.1186/s12934-019-1176-z)
Supplement: Supplementary file 1 — Additional file 1: Figure S1. Opp system in Bacillus, oligopeptide ABC transporter family. A, an extracellular ligand-binding lipoprotein (OppA), two transmembrane proteins (OppBC) that form a membrane-spanning pore, and two cytoplasmic ATPases (OppDF) that drive the transport of the peptide into the cell; B, 3D structure of OppA protein; C, Identification of oligopeptide transporter systems in Bs-916. [file 12934_2019_1176_MOESM1_ESM.docx]

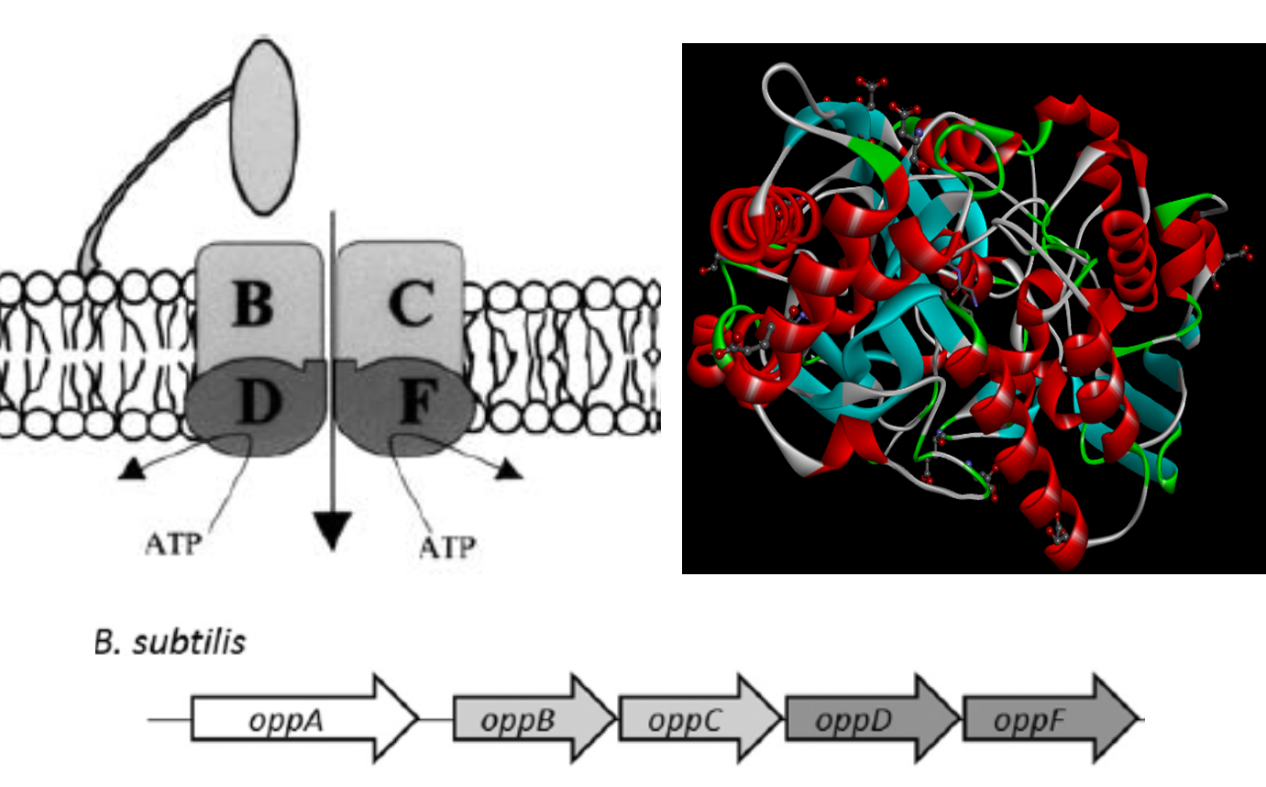


**A B**

**C**

Figure S1. Opp system in *Bacillus*, oligopeptide ABC transporter family. A, an extracellular ligand-binding lipoprotein (OppA), two transmembrane proteins (OppBC) that form a membrane-spanning pore, and two cytoplasmic ATPases (OppDF) that drive the transport of the peptide into the cell; B, 3D structure of OppA protein; C, Identification of oligopeptide transporter systems in Bs-916.
